# Aquaporins in development – a review

**DOI:** 10.1186/1477-7827-3-18

**Published:** 2005-05-11

**Authors:** Huishu Liu, E Marelyn Wintour

**Affiliations:** 1 Guangzhou Obstetric and Gynecology Institute, Second Municipal Hospital of Guangzhou, Guangzhou Medical College, Guangzhou, PR China; 2 Department of Physiology, Monash University, Clayton, Victoria, 3800, Australia

## Abstract

Water homeostasis during fetal development is of crucial physiologic importance. It depends upon maternal fetal fluid exchange at the placenta and fetal membranes, and some exchange between fetus and amniotic fluid can occur across the skin before full keratinization. Lungs only grow and develop normally with fluid secretion, and there is evidence that cerebral spinal fluid formation is important in normal brain development. The aquaporins are a growing family of molecular water channels, the ontogeny of which is starting to be explored. One question that is of particular importance is how well does the rodent (mouse, rat) fetus serve as a model for long-gestation mammals such as sheep and human? This is particularly important for organs such as the lung and the kidney, whose development before birth is very much less in rodents than in the long-gestation species.

## Introduction

There are, at present, eleven known members of the mammalian aquaporin gene family, which encode proteins which function as membrane channels, for water alone (AQP0,1,2,4,5,8,10), or for water plus small molecules, mostly glycerol and urea (AQP 3, 7, 9), or nitrate (AQP 6) [1-5]. In some cases the aquaporin is constitutively present in the cell membrane (e.g. AQP1,3 in red cell membrane, AQP1 kidney). However, in other cases the aquaporin resides in intracellular vesicles, and is trafficked to the membrane upon appropriate stimulation e.g. AQP 2 in collecting duct cells, after vasopressin exposure [6]; AQP1 in cholangiocytes with secretin stimulation [7]; AQP 8 in hepatocytes, after glucagon treatment [8,9]; aquaporin 5 in rat parotid, with muscarinic stimulation [10]. These aquaporins subserve the rapid transport of fluid across epithelial and endothelial cells, but are also found in other tissue types, such as muscle and nerve cells. In general the water channels are 'open' but there is some evidence that 'closure' can be induced by a specific treatment.

During development there are some unique fluid compartments (amniotic, allantoic fluids, lung liquid) and the functions of some organs, such as the kidney, differ from the function in the adult, as discussed below. Although some insights into the developmental roles of aquaporins might be obtained from the study of mice with deletions of various aquaporin genes, this is complicated by the facts that either much of normal organ development occurs postnatally in the rodent, rather than prenatally in the human (e.g.kidney), chick (brain) or the ontogeny of aquaporins differs significantly in the rodent organ (e.g.lung) from that in long-gestation species such the sheep [11]. In addition, the fetus contains a higher percentage of water than does the adult, and organs such as the brain are more vulnerable to excess water loss which might occur in the premature neonate, due either to immaturity of the skin permeability barrier, or to immaturity of the water-retaining functions of the kidney. The role of aquaporins in fluid balance during fetal development is beginning to be explored.

## Placenta and fetal fluid compartments

Amniotic fluid surrounds the developing fetus and is essential for normal morphological development. Inputs into amniotic fluid include the dilute fetal urine and the isotonic lung liquid, and pathways of exit of fluid include fetal swallowing, and transmembrane fluxes [12,13]. Thus abnormalities of amniotic fluid volume (oligo- and polyhydramnios) can result from abnormalities in fetal renal function, and oligohydramnios can be corrected, to some extent, by increase in maternal hydration [14,15]. Under normal circumstances the fetal fluid osmolality follows that of the mother, and fluid exchange occurs across the placenta, as well as across the amnion/chorion [16].

Before implantation the conceptus develops into a blastocyst, composed of the inner cell mass, and a fluid filled cavity surrounded by trophoectoderm epithelium. In the mouse aquaporins 3, 8, and 9 have been found to be expressed at this time, AQP3 and AQP8 being predominantly in the basolateral membranes of the trophoectoderm, and AQP9 in the apical membrane [17]. The trophoectoderm gives rise to the placenta and chorion; aquaporins 1, 3, 8 and 9 are water channel genes previously reported to be in the placenta and/or chorion of the human and sheep [18-21]. AQP1 has also been reported to be in the chick chorioallantoic membrane [22]. AQP1 is in the vasculature and AQP3 and 9 are in the apical membranes of human and ovine term placenta and chorion. The polarity of the AQP 8 has not yet been determined [18-20]. Recently we reported that AQP8 mRNA was also found in the ovine placenta [23].

From 45 d gestation (term is ~150 days), AQP3, functioning both as a water and urea channel, and expressed in the trophoblast epithelial cells, is the major AQP, which increases throughout gestation, and is quantitatively the most highly expressed AQP gene in the ovine placenta. The permeability of the ovine placenta to urea increases markedly after ~100 days of gestation, coordinately with a sharp increase of AQP3 expression in the placenta at this time.

Similarly, AQP8, which is expressed in the trophoblast epithelial cells and membrane epithelial cell [24], is also present at significant levels from 45 d gestation.

In sheep, the placenta ceases growth close to mid-gestation, despite the dramatic increase in the fetal weight during the last half of gestation [25]. To maintain fetal growth, there is a requirement for increased fluid transfer to the conceptus. The presence of substantial expression of water channel proteins in the placenta correlates well with the placental transfer of fluid. It was not possible to compare expression at the protein level as large quantities of AQP1 and AQP3 protein, in the maternal red cell membranes present in the haemophagous zone of the ovine placenta [26]. Thus comparison at the mRNA level is the only feasible one that can be made.

## Kidney function in the fetus

The fetal metanephric kidney produces a relatively large volume of dilute urine, essential for the maintenance of amniotic and (in some species) allantoic fluid volumes. In the most common animal model (sheep) used for the study of fetal renal function it has been shown that the volume of urine production is 0.3 l/kg/d compared with 0.02 l/kg/d in the adult sheep. This occurs in spite of a glomerular filtration rate which is approximately one third of adult values, and is due to both a decrease in total sodium reabsorption (95 % in the fetus vs 99% in the adult) and to absence of significant concentration of the urine. In the unstressed ovine fetus the urine osmolality is always less than 200 mosmoles.kg water, and may be as low as 60 [27].

## Aquaporins in development – kidney

In the adult kidney the bulk of the filtrate (81%) is reabsorbed in the proximal tubule and descending limb of the loop of Henle, where AQP 1 is expressed. AQP1 is also expressed in the nonfenestrated descending vasa recta which are thought to be important for the establishment of the hypertonic environment of the medulla. In the mouse with the AQP1 gene deleted there is a lowered capacity to maximally concentrate urine [28]. However, the major concentration of urine depends on the presence of aquaporin 2 in the apical membranes of the principal cells of the collecting duct. This water channel protein resides in sub -membraneous vesicles in the absence of action of circulating vasopressin. Under the stimulus of increased vasopressin second messenger systems are activated which result in the phosphorylation of the vesicular AQP2 and transport and insertion into the apical membranes. Without this water channel it is impossible to reabsorb water in the medulla, even when an adequate osmotic gradient exists [2]. In many situations in which polyuria/concentrating defect occurs (potassium deficiency, lithium levels greater than 0.3 mmol/l, hypercalcemia, low protein diet among others) it can be linked to low levels of AQP2 [2]. The water absorbed via AQP2 in the apical membrane leaves the cell via aquaporins 3 and 4 which are constitutively expressed in the basolateral membranes of these cells [2]. In mice lacking expression of the AQP1 gene there is polyuria, and failure to be able to concentrate urine normally [28], and a similar urinary concentrating defect is seen in the rare humans who lack AQP1 [29]. A milder urinary concentrating defect is seen in transgenic mice lacking AQP4 [30]. This maybe because AQP3 is colocalised with AQP4 on the basolateral membrames of collecting duct principal cells, but when AQP3 is deleted a poyuria with a severe concentrating defect occurs [31]. AQP3 levels are regulated to some extent by vasopressin, as are those of AQP2, but are also regulated by aldosterone and the cystic fibrosis transmembrane conducting factor (CFTR) [2,32-34].

Metanephric kidney development varies in different species, being complete before birth in human and sheep, but not until substantially after birth in pigs, mice and rats. The ontogeny of some renal aquaporins has been examined in rats, sheep and humans. In the rat there is very little mRNA for AQP1 detected by Northern blotting or RNase protection, in the kidney, until a few days before birth [35,36]. However, there is some protein detected, by immunohistochemistry in the capillaries at the nephrogenic zone-medullary border by day 16. From day 17 the arcuate arteries are labeled, and, indeed the descending vasa recta are strongly labeled as they develop fully until 21 days post partum [37]. In contrast, in the sheep and human kidneys, AQP 1 mRNA, and protein are detected before mid-gestation (12 /40 weeks, human; 41/150 days, sheep) though the levels are just below 50% of adult levels even at term [38,39]. Levels of expression can be increased by both glucocorticoid and angiotensin II treatment of the fetus, both probably due to maturation of the kidney and longer proximal tubules which develop with treatment [39]. Adult levels are achieved after 15 months in the human, or 6 weeks in the sheep.

Aquaporin 2 (AQP2) is low at birth in the rat, but plateaus by 4 weeks post-partum [40]. Later studies showed it was present by Day 18 of fetal life and started increasing by day 3 post-natally [41]. In the sheep, at the beginning of the last third of gestation (100/150 d) the level of AQP2 mRNA is 17% of the adult, and near term it is still only ~40% of the adult [42]. This correlates with reduced sensitivity of the fetal kidney to infused arginine vasopressin – at 100 days the plasma AVP concentration has to be raised to 16 pg/ml to achieve negative free water clearance, whereas close to term a level of 2 pg.ml is effective [43]. This is still a much higher level than required in the adult sheep, and so the fetal kidney resembles that of a subject with nephrogenic diabetes insidipus, due to inadequate expression of AQP2. The human fetal kidney also has a low level of AQP2 during the last half of gestation, and premature neonates produce dilute urine for many weeks [38,44]. AQP2 protein does appear in the urine [45], and there is a low level in the urine of premature neonates [46]. However the concentration of AQP2 protein in the urine of premature neonates did not correlate well with changes in urine osmolality, suggesting that it did not serve as a good marker of AVP function in the human premature neonate [47]. Fetal renal AQP2 levels can be increased by angiotensin II infusion, which is a real up-regulation of gene expression, and similar to the up-regulation of vasopressin V2 receptor seen with angiotensin II infusion in adult rats [48].

There has been one study of AQP3, in fetal kidneys, suggesting it is there by day 18 in the rat [41]. The level of AQP4 protein labeling is very weak in the rat kidney 3 days after birth [36].

The low level of AQP2 expression, however, seems to be the major factor in allowing the production of a large volume of hypotonic urine to be formed, and this is essential for the maintenance of adequate volumes of amniotic fluid.

## Lung liquid

During fetal life, the future airways of the lung are filled with a liquid that plays a crucial role in the growth and development of the lungs by maintaining them in an expanded state. Lung liquid is secreted across the pulmonary epithelium into the lung lumen due to the osmotic gradient established by the net movement of Cl^- ^in the same direction. It is not known exactly when lung liquid secretion begins, but fluid is present by mid-gestation in fetal sheep and is secreted at 2–4 ml/kg/h between 120 days of gestation and term (~150 d). Fetal lung liquid exits the lungs via the trachea, whereby it is either swallowed (approximately 50%) or passes directly into the amniotic sac, where it contributes to amniotic fluid volume [49].

If the fetal trachea is obstructed, which prevents the outward flow of lung liquid, the fetal lung expands with accumulated liquid. This is a potent stimulus for fetal lung growth and also greatly reduces the proportion of type-II alveolar epithelial cells (AECs). Lung liquid drainage on the other hand, deflates the lung, causes lung growth to cease, but increases the proportion of type II AECs, possibly via type-I to type-II cell differentiation [50]. As a result it is now widely recognized that the degree to which the fetal lungs are expanded by lung liquid, determines the growth and structural development of the lung, as well as the differentiated state of type-I and type-II AECs [49]. Despite the importance that lung liquid plays in the development of the lung, the factors controlling the movement of liquid across the pulmonary epithelium have not been fully explored. Furthermore, the effective clearance of lung liquid at birth is vital to allow the entry of air into the lungs with the onset of respiratory gas exchange. This process is largely dependent on the capability of the epithelium to reabsorb large quantities of water.

## Aquaporins in development – lung

At least four AQPs (AQP 1, 3, 4 and 5) are expressed in the lungs of various species, including humans, rats, mice and rabbits, although some discrepancies exist in the specific sites of distribution of these proteins. (Table 1 near here) In all species described so far (human, rat, mouse), AQP1 is expressed in the apical and basolateral membrane of the microvascular endothelium and decreased pulmonary vascular permeability has been shown in AQP1-null humans [3]. AQP3 is expressed in the basolateral membrane of basal cells of the tracheal epithelium and in submucosal gland cell membranes in rodents, but is also found in bronchioles (apical membrane) and type-II alveolar epithelial cells of adult humans [51]. AQP4 is present in the basolateral membrane of columnar cells in bronchi and trachea of rats but is also found in type-I AECs in humans. AQP5 is expressed in the apical membrane of type-I AECs and the apical plasma membranes of the secretory epithelium in upper airway and salivary glands [3]; it has also been detected in type-II AECs in mice [52]. These data are summarized in Table 2.

**Table 1:** Species variations in Aquaporin Distribution in Lung

Species	Sheep	Human	Rat	Mouse
Bronchus	AQP1,3,4,5	AQP1,3,4,5	AQP1,3,4,5	AQP1,3,4,5
Bronchioles	AQP1,3,4	AQP1,3	?	?
Alveoli	AQP1,5	AQP1,3,4,5	AQP1,3	AQP1,3

**Table 2:** Aquaporins in lung cell types

Bronchus	
Superficial Epithelium	AQP5 (Apical), AQP4 (Basolateral)
Basal Cells	AQP3
Submucosal Glands	AQP5 (Apical), AQP3,4 (Basolateral)

Bronchioles	
Pseudostratified	AQP3 (Apical), AQP4 (Basolateral)

Alveolar Cells	
Type I	AQP5 (Apical), AQP4 (Human only--?)
Type II	AQP5 (Mouse only, apical)
	AQP3 (Human only, basolateral)

## Ontogeny of lung AQPs

In mice very low levels of AQP5 mRNA were detected before birth [53,54]. The ontogeny of the AQPs has also been described throughout development in rats, but only AQP1 and a small amount of AQP4 were detected before birth [55-58]. Furthermore, little is known of the physiological factors controlling AQP1 mRNA expression before birth, although its expression (and protein levels) is increased in the lungs of fetal and neonatal rats following treatment with synthetic glucocorticoids [55,58]. In one study [58], but not in another [55], AQP4 was increased by corticosteroids. In the same study [58], β-adrenergic agents also increased AQP4. Although AQP5 protein was almost undetectable in lung tissue homogenates at E21 and PN1, a strong signal was detected at PN2 [55], indicating that the accumulation of AQP5 protein in the rat lung is predominantly postnatal. Indeed, AQP5 protein levels in lung tissue increased twenty-fold to PN14 and then increased a further ten-fold from PN14 to adult. In contrast to AQP1, AQP5 is not influenced by corticosteroids in rats, which is consistent with the finding that AQP5 protein predominantly accumulates in the lung postnatally. Similarly, AQP3 protein levels were undetectable in fetal lung tissue and then were only detected in the trachea of postnatal animals well after the time of birth. AQP4 protein seemed to be present transiently at PN2 in peripheral lung membranes and only appeared by PN12 in the trachea of rats

In a recent study we have shown that the mRNAs for at least four AQPs (1, 3, 4 and 5), as well as their respective proteins, are present in the ovine fetal lung well before birth [11]. For AQP1 and AQP5, the level of mRNA expression in the fetal lung exceeded that of the adult lung. Furthermore, we have shown that cortisol infusions significantly up-regulated the expression of AQPs 1 and 5, whereas increases in fetal lung expansion, induced by tracheal obstruction (TO), significantly decreased AQP5 mRNA levels in fetal lung tissue. Although AQP5 protein levels did not appear to decrease with TO, measurable changes in AQP5 levels in whole lung tissue is likely to be complicated by the localisation of this protein to multiple cell types within the lung. These findings indicate that factors known to regulate fetal lung growth and maturation as well as fluid secretion, also regulate the expression of AQPs 1 and 5. This suggests that there are physiological roles for some lung aquaporins before birth.

In conclusion, we have shown that the lung of a long-gestation species, such as sheep, expresses both the mRNA and protein of the four typical lung AQPs, beginning well before the expected time of birth. Furthermore, we found that the expression of some, particularly AQP5, is altered by factors known to regulate fetal lung growth and development and parallel changes in fetal lung liquid secretion rates in different animal models. Our findings suggest that gene knock-out studies in mice, in which there is little lung expression of AQPs in fetal life, might not give a realistic picture of the role of AQPs during fetal life in long-gestation species. We predict that these AQPs are also expressed well before birth in the human fetal lung and are also differentially regulated by factors known to influence fetal lung development. As lung liquid is secreted, at least in part, into amniotic fluid, the lung aquaporins are also then implicated in amniotic fluid regulation.

## Skin

The skin of the adult 70 kg man normally contains about 7 l of fluid, about 50% of which is interstitial [59]. The fluid is stored in the dermis associated with hyaluronic acid, glycosaminoglycans and proteoglycans, and helps to determine the turgor, distensibility and elasticity of the skin. The major barrier to water loss from the skin is the superficial stratum corneum – flattened dead corneocytes [60]. Below this are the keratinocytes, which express the gene for aquaporin 3, particularly in the basal and intermediate layers [61-63]. Aquaporin 3 is a membrane protein which increases the permeability to water, urea and glycerol. When the gene is deleted in the mouse the skin has decreased hydration but grossly normal morphology [62]. The reduction in skin elasticity, as well as the delay in recovery of barrier function after tape stripping, were thought to be related to the deficiency in glycerol transport which occurred in the AQP3 deficient mice [64]. This was further supported by the reversal of these deficits by glycerol replacement [65].

## Aquaporins in development – skin

In the human fetus there is a double layer of epidermal cells by 4 weeks; the stratum corneum begins to develop by 24 weeks, and is generally well developed by 34 weeks. [60]. Barrier function, which is conferred by the stratum corneum, of cornified cells and extracellular lipid, can be measured by transepidermal water loss (TEWL), and generally forms late in gestation in mice, rats, rabbits and humans [66,67]. Amniotic fluid, particularly early in pregnancy, is very similar in composition to fetal extracellular fluid, and it is quite likely that here is fairly free exchange across the fetal skin, particularly in the first half of gestation [68]. Even in species such as the sheep, which develop substantial wool covering in the last third of gestation, there is substantial exchange of fluid and electrolyte across the skin until relatively late in development [69]. There is also substantial expression of AQP3 in mid-gestation ovine fetal skin. Preterm infants are at risk of dehydration because of very large TEWL [70]. In fetal rats the TEWL is high at day E18, and there are higher levels of AQP3 mRNA in the fetal than in the adult skin [71].

## Aquaporins in the heart – changes with intrauterine growth retardation (IUGR)

Aquaporin 1 mRNA was found in rat heart [72,73]. Most of the AQP1 expression was thought to be in the blood vessels, although the there was a substantial amount in a sub-sarcolemnal caveolar membrane in the rat heart, and changes in the osmotic environment caused reversible changes in the membrane localization of AQP1 [74]. Recently it was found that the human heart contained both AQP1 and AQP4, but not AQP8 [75]. AQP1 co-localised with vinculum, a t-tubule component, and caveolin 3, whereas AQP4 was found in the nuclear membrane of human cardiac myocytes.

Caveolin-3 is a marker for the caveolae – the specialised areas of cell membrane in which a number of receptors cluster [76]. Some of these receptors are known to play a role in the proliferation of cardiac myocytes in the embryonic and early post-natal life [77-80].

Based on studies in isolated rabbit hearts, it was concluded that water permeabilitity values were much lower than expected if a functioning aquaporin were present [81]. In a more recent study of the osmotic transient responses of isolated adult rabbit hearts [82] it was estimated that 28% of the transcapillary water flux going to form lymph was through aquaporin channels in the capillaries, but they did not make any histological studies on the cardiocytes. It would have been very interesting to have had immunohistochemistry for AQP1, at least, on these hearts.

During development AQP1 was found in the endocardium of the sheep fetal heart at a very early stage [83]. Later in gestation one report suggested that total cardiac AQP1 levels reflected predominantly vascular sites, and that the total amount could be increased by fetal anemia [84].

Using RNase protection assay only AQP1 (but not AQPs 2,3,4,5) was detected in rat heart [72], however with RT-PCR some AQP8 mRNA was detected in mouse heart [85]. AQP 1 was reported to be present in fetal rat hearts from day E14 with lower level present in the myocardium than in the endothelial cushions, primordial valves, and septa [35]. Cardiac expression of AQP1 decreased, but did not disappear, after birth [35].

In a recent study we showed that the small hearts of late gestation growth-retarded ovine fetuses had significantly reduced expression of AQPs 1,3,4 but not AQP8 [86]. It was not possible to ascertain the different contributions of cardiac muscle and blood vessels to this reduced expression. In the fetal sheep heart at mid-gestation, all the myocytes are uninucleated and can divide, but by 135 days more than 50% of the myocytes are binucleated, and terminally differentiated [87]. When growth retardation occurs in the fetal heart, we postulated that it might have occurred by 'slowing down' of cell division resulting in a greater proportion of uninucleated cells in late gestation. In order to see whether AQP1 can be a marker of cardiac myocyte differentiation, we measured the AQP1 mRNA concentration in hearts from fetuses in which cardiac myocyte counts had been performed previously. Our results show that the level of AQP1 mRNA expression did not change significantly at any point during gestation, suggesting that it could not be used as a marker of cardiac myocyte differentiation. Thus the heart is different from vascular smooth muscle.

In conclusion, we demonstrated that the AQP1/3/4/8 are present in the late gestational fetal heart. The low-dose dexamethasone treatment, administered early in gestation, down regulated the expression of AQP1/3/4 in the late gestation fetal heart. In most studies of experimentally induced fetal growth retardation some organs, eg the brain and adrenal gland are 'spared', but others, such as the heart, are reduced in size in proportion to the overall decrease in body size [88]. There are a number of genes which have been implicated in cardiac myocyte growth, including mineralocorticoid and glucocorticoid receptors, angiotensin II receptors, and local cardiac angiotensinogen [89-96]. However, the mRNA for none of these was affected in the hearts of the IUGR fetuses.

There is evidence in the literature suggesting that fetuses suffering from severe intrauterine growth retardation (IUGR) show a progressive impairment of cardiac function, as demonstrated by reduced peak velocities at outflow tracts, decreased cardiac output and abnormal venous flow patterns [95-98]. Furthermore, in growth-retarded human fetuses the ventricular ejection force was equally decreased in both ventricles [95]. Studies in the adult offspring of rats subjected to prenatal protein restriction, which caused IUGR, demonstrated higher incidence of cardiac arrhythmias and raised diastolic blood pressure [97].

The exact function for AQP1 in cardiac muscle is unknown. As it is a pure water channel one would suspect that it could regulate the rate at which cells might swell in osmotic stress, such as encountered in myocardial ischemia [98]. Such osmotic swelling is predicted to shorten the action potential, thus modulating the excitability of the heart. It is known that cell swelling inhibits the action of some antiarrhythmic drugs [98]. AQP 4 is well established as a component of skeletal fast-twitch fibres [99] and the level of AQP4 is decreased by muscle denervation [100]. In mice which are dystrophic due to dystrophin gene knock-out (mdx mice) AQP4 mRNA levels remain the same as controls, but the protein levels decrease by 90% [101]. However, in patients with Duchenne muscular dystrophy both the mRNA and protein of AQP4 are reduced in myofibers [102]. Taken together it is attractive to propose that AQP's play a role in the cardiac myocyte contraction allowing therefore normal cardiac function.

## Brain-central, nervous system, eye, ear-fluid compartments

In the adult brain fluid balance is critical, as the inflexible bony skull does not permit big variations in total brain volume without risking severe damage. The extracellular fluid of the brain is specialized as cerebrospinal fluid, with a composition different from that of normal extracellular fluid, as a result of the development of the 'Blood-brain barrier'. There is now increasing evidence that cerebrospinal fluid plays an important part in the correct development of the brain [103,104]. Specialised fluid compartments are also vital to the normal functioning of the sensory organs – the eye and the ear [105,106]. In the eye fluid movements are important for the regulation of intraocular pressure, the maintenance of transparency of the lens, and retinal signal transduction [106]. The fluids of the inner ear, endolymph and perilymph, have at least two roles – to transduce the signal to the cochlear and vestibular hair cells, and to participate in the ionic exchanges between fluid and hair cells [106]. The endolymph is a potassium-rich extracellular fluid, whereas the perilymph has a composition closer to that of extracellular fluid [107]. It is well-known that vestibular functions can be altered by a number of peptide e.g arginine vasopressin, and steroid hormones [108-110], which act by changing composition, and maybe the volume, of the endolymph.

A number of aquaporins have been found in the central nervous system – AQPs 1,4,5,9 [111,112]. AQP1 is found on the apical membrane of the epithelial cells of the choroid plexus. AQP 4,5, and 9 are found on glia/astrocytes particularly in the region of subpial vessels and near the ventricles. Of these it seems that AQP4 provides the principal route for water transport in astrocytes [113]. Glial cells are indispensable for regulating ionic homeostasis, particularly in aspirating the excess extracellular potassium which occurs after neural excitation [107]. It is of interest that in the specialized glial Muller cells of the eye, there is a close correlation between concentrations of the potassium channel, Kir4.1 and AQP4 levels [114], and retinal function is mildly impaired in mice lacking AQP4 [115]. The absence of AQP4, in the brain, paradoxically, in the genetically-engineered 'knock-out' mouse, reduces the swelling seen with hyponatremia [116]. The distribution of AQP4 protein is disrupted in the dystrophin-deficient mdx mouse, in which a 60% reduction occurs in the amount of AQP4 in the perivascular glial processes, which are swollen and contain debris [101,104]. In these mice the there is a marked reduction in the amount of AQP4 in the astroglial feet surrounding capillaries, and at the glia-limitans, and a significant delay in the in the development of brain edema induced by systemic hyponatremia [117]. The protein, alpha syntrophin, is associated with the dystrophin, and also important for the anchoring of the AQP4 in the cell membrane [118]. In mice lacking the alpha-syntrophin gene the there is also a marked loss of AQP4 from perivascular and subpial membranes, but no decrease in other membrane domains, and brain edema was attenuated when transient ischemia was induced [119]. All of this evidence suggests that any inhibitor of AQP4 expression may have therapeutic benefits in the treatment of brain edema [111,112].

The ontogeny of AQP4 in the cerebellum coincides with the development of the blood brain barrier in rat and chick. [120,121]. In the rat brain there is no AQP4 before birth [122] and only 2% of the adult level one week after birth. The level doubles in the next week, and reaches 63% of adult levels by nine weeks. In contrast, the chick brain, has a much better level of AQP4 at birth and a more mature blood-brain barrier [121]. This has not yet been studied in the human, but one would expect that the very premature baby would have little barrier protection.

In the ear of the adult rat mRNA for aquaporins 1,2,3,4,5,6 have been found [109], whereas AQP7 and AQP9 were also detected in the adult mouse, but at relatively low levels [122]. Aqp1 is strongly expressed in the non-epithelial stria vascularis [123] and can be up-regulated, in a dose-dependent fashion, by intra-tympanic injections of dexamethasone [109]. AQP1 was detected at the earliest day studied, E14, in mice but in much lower concentrations than those found in the adult ear [122].

AQP2 mRNA, at 10% of the levels found in kidney, is found in rat and mouse ear [124]. It is in structures bordering the endolymph – Reissner's Membrane, Organ of Corti, sulcus cells, and spiral limbus. Treatment of rats with arginine vasopressin caused a doubling of AQP2 mRNA in the cochlea and endolymphatic sac [125,126], and the authors suggested that overexpression of AQP2 might be involved in the formation of endolymphatic hydrops. During development of the ear in the mouse AQP2 was expressed diffusely in the early otocyst at embryonic days 12,13 but the expression became more restricted by days 15–18 [127].

Quantitatively the most important aquaporin expressed in the ear is AQP4, and it is expressed in Hensen's cells and inner sulcus cells and Claudius cells, which are all supporting cells of the Organ of Corti [128]. In the vestibular end organs it was in the cristae and maculae. It also occurred in the central part of the cochlear and vestibular nerves. In mice lacking AQP4 expression there is a moderate impairment of hearing [129], but no conduction abnormality was detected in neural signals [130]. AQP4 was detected by E14 in the developing mouse ear, and the level was increased ~100 fold during after birth and continued to increase through post-natal day 15 and even further in the adult [122].

AQP3 was found by one group [122] in the spiral ligament of the mouse cochlea, near where the basilar membrane anchors, and in cells bordering the inner spiral tunnel. In the vestibular system it was in sub-epithelial fibrocytes in the saccule, but not in the utricle. There was a moderate increase in AQP3 from day E14 to adult.

All these results in rodents are tantalizing, and it will be very interesting to see the ontogeny of brain and sensory organ aquaporins in the primate/human. It is expected that significant expression of these water channels will be seen well before birth, as is the case for the lung in long-gestation species [11].

## Conclusion

Much information on the role of various members of the mammalian aquaporin family of water channels has been gained in the relatively short time since Peter Agre and his colleagues described the Channel-forming integral membrane protein of the red blood cell of 28 kD (CHIP28), [1], and justifiably earned the 2003 Nobel Prize for Chemistry. Some exciting new studies are suggesting that AQP1 may have roles hitherto unsuspected – evidence has been obtained supporting a role for AQP1 in angiogenesis, particularly in wound healing, organ regeneration and possibly in tumour spread [131]. The limited information that exists on the ontogeny of these proteins in various organs and tissues suggests that there are many more important findings to be made on their roles in the development of the embryo and fetus.
